# *Epilepsia Open*—April 2024 announcements

**DOI:** 10.1002/epi4.12932

**Published:** 2024-04-01

## ILAE Congresses

ILAE School on Neuroimaging 2024 **(AMIE & SuSIE 2024)**

15–18 May 2024

Potsdam, Berlin & Online

XIII Congreso Latinoamericano de Epilepsia

15–18 June 2024

Santo Domingo, República Dominicana

6th ILAE School on Advanced EEG and Epilepsy in Dianalund

20–27 July 2024

Dianalund, Denmark

15th European Epilepsy Congress

7–11 September 2024

Rome, Italy

14th International Summer School for Neuropathology and Epilepsy Surgery

19–22 September 2024

Erlangen, Germany

15th Asian & Oceanian Epilepsy Congress

November 2024

New Delhi, India

7th East Mediterranean Epilepsy Congress

14–16 November 2024

Manama, Bahrain

36th International Epilepsy Congress

30 August–3 September 2025

Lisbon, Portugal

## ILAE Webinars

Epilepsy Educational Webinar

2 April 2024

Webinaire éducatif sur l'épilepsie en français

16 April 2024

Living with Epilepsy: Conversation between a patient and a psychologist

25 April 2024

Epilepsy Educational Webinar

26 April 2024

Climate Change and Epilepsy: The impact on incidence of epilepsy on shifting patterns of vector borne disease

9 May 2024

ILAE e‐Forum: Beyond the seizures in childhood onset epilepsies

10 June 2024

ILAE e‐Forum: Co‐morbidities and mental health in adults with epilepsy

1 August 2024

ILAE e‐Forum: Reducing epilepsy related mortality

23 September 2024

ILAE e‐Forum: Optimal timing for epilepsy surgery

25 November 2024

## Other Congresses

2nd International Conference on Artificial Intelligence in Epilepsy and Neurological Disorders

1–4 April 2024

Park City, Utah, USA

Curso taller internacional de electroencefalografía básica y avanzada

5–6 April 2024

Online

The Ketogenic Diet Trans‐Caribbean Workshop

4–5 April 2024

Kingston, Jamaica

The Holistic Approach to Managing Epilepsy: Clinical care, education, and research

6–7 April 2024

Kingston, Jamaica & Online

Pre‐Colloquium Teaching Course ‐ Continuous EEG in ICU

7 April 2024

London, United Kingdom

9th London Innsbruck Colloquium on Status Epilepticus and Acute Seizures

8–10 April 2024

London, United Kingdom

The Riddles of Childhood Epilepsy and the Road Ahead: Honouring the legacy of Carol and Peter Camfield

8 April 2024

Toronto, Ontario, Canada

EPIPED Course: Treatment strategies in pediatric epilepsies

10–13 April 2024

Girona, Spain

Paros Epilepsy

22–26 April 2024

Paros, Greece

6th Qatar International Epilepsy Course

25–27 April 2024

Qatar

Seventeenth Eilat Conference on New Antiepileptic Drugs and Devices **(EILAT XVII)**

5–8 May 2024

Madrid, Spain

International Conference for Post‐Traumatic Epilepsy

15–18 May 2024

Milan, Italy

13th World Congress for NeuroRehabilitation

22–25 May 2024

Vancouver, Canada

Third International Workshop on High Frequency Oscillations in Epilepsy

23–25 May 2024

New York, NY, USA

SNS Annual Meeting 2024

6–7 June 2024

Basel, Switzerland

62. Jahrestagung der Deutschen Gesellschaft für Epileptologie

12–15 June 2024

Offenburg, Germany

29th Korean Epilepsy Congress

21–22 June 2024

Seoul, South Korea

The Comorbidities of Epilepsy

21 June 2024

London, UK & Online

10th Congress of the European Academy of Neurology

29 June–2 July 2024

Helsinki, Finland

20th Advanced San Servolo Epilepsy Course

21 July–1 August 2024

San Servolo (Venice), Italy

XLVI Reunión Anual del Capítulo Mexicano de la Liga Internacional Contra la Epilepsia

6–10 August 2024

Monterrey, Nuevo León, Mexico

10th Eilat International Educational Course: Pharmacological Treatment of Epilepsy

15–20 September 2024

Jerusalem, Israel

16th International Epilepsy Course and Colloquium

17–20 September 2024

Frankfurt am Main, Germany

Epilepsy Society of Australia Annual Scientific Meeting 2024

6–8 November 2024

Hobart, Tasmania, Australia

